# On the Stability of Deinoxanthin Exposed to Mars Conditions during a Long-Term Space Mission and Implications for Biomarker Detection on Other Planets

**DOI:** 10.3389/fmicb.2017.01680

**Published:** 2017-09-15

**Authors:** Stefan Leuko, Maria Bohmeier, Franziska Hanke, Ute Böettger, Elke Rabbow, Andre Parpart, Petra Rettberg, Jean-Pierre P. de Vera

**Affiliations:** ^1^German Aerospace Center, Research Group “Astrobiology”, Radiation Biology Department, Institute of Aerospace Medicine Köln, Germany; ^2^German Aerospace Center, Institute of Optical Sensor Systems Berlin, Germany; ^3^German Aerospace Center, Institute of Planetary Research Berlin, Germany

**Keywords:** Raman spectroscopy, *Deinococcus radiodurans*, deinoxanthin, Mars

## Abstract

Outer space, the final frontier, is a hostile and unforgiving place for any form of life as we know it. The unique environment of space allows for a close simulation of Mars surface conditions that cannot be simulated as accurately on the Earth. For this experiment, we tested the resistance of *Deinococcus radiodurans* to survive exposure to simulated Mars-like conditions in low-Earth orbit for a prolonged period of time as part of the Biology and Mars experiment (BIOMEX) project. Special focus was placed on the integrity of the carotenoid deinoxanthin, which may serve as a potential biomarker to search for remnants of life on other planets. Survival was investigated by evaluating colony forming units, damage inflicted to the 16S rRNA gene by quantitative PCR, and the integrity and detectability of deinoxanthin by Raman spectroscopy. Exposure to space conditions had a strong detrimental effect on the survival of the strains and the 16S rRNA integrity, yet results show that deinoxanthin survives exposure to conditions as they prevail on Mars. Solar radiation is not only strongly detrimental to the survival and 16S rRNA integrity but also to the Raman signal of deinoxanthin. Samples not exposed to solar radiation showed only minuscule signs of deterioration. To test whether deinoxanthin is able to withstand the tested parameters without the protection of the cell, it was extracted from cell homogenate and exposed to high/low temperatures, vacuum, germicidal UV-C radiation, and simulated solar radiation. Results obtained by Raman investigations showed a strong resistance of deinoxanthin against outer space and Mars conditions, with the only exception of the exposure to simulated solar radiation. Therefore, deinoxanthin proved to be a suitable easily detectable biomarker for the search of Earth-like organic pigment-containing life on other planets.

## Introduction

The search for evidence of extant or extinct life on Mars by *in situ* investigations began in 1976 with the landing of the Viking spacecraft (Klein, 1999) and continues today with the curiosity rover investigating whether life was ever present on Mars. The climatic history of Mars can be divided into three main eras, beginning with a water-rich epoch (Noachian; <3.95–3.7 billion years), followed by a cold and semi-arid period (Hesperian; 3.7–2.9 billion years) and transitioning into present-day arid and cold desert conditions (Amazonian; 3.1 billion years to present) (Fairén et al., 2010). Concerning the habitability of Mars, these eras also represent three stages of habitability (Fairén et al., 2010). The Noachian era may represent a potentially habitable epoch, when basic requirements for life as we know it, such as water and energy were present on Mars. The Hesperian era was possibly very challenging for life with liquid solutions evaporating and during the Amazonian era, the surface of Mars has become, except for some subsurface niches (de Vera et al., 2014; Schirmack et al., 2014), uninhabitable to life as we know it. Although there is no definitive proof that life existed on Mars, the possibility is intriguing and the search for signs of extinct or extant life is one of the main focuses of Astrobiologists.

Whenever life-forms were present in an environment, they often leave traces of their former presence in the form of biomarkers. A biomarker can be defined as a chemical species or pattern which is uniquely derived from a living organism (Edwards et al., 2014). Among the most widespread and stable bacterial biomarkers is the class of hopanes (Toporski and Steele, 2002), which are pentacyclic organic compounds found in bacterial membranes where they are used to control cell membrane permeability and aid in their adaptation to extreme environmental conditions (Edwards et al., 2011). Another important group of biomarkers are carotenoids, which can serve as accessory pigments in the light-harvesting complexes of photosynthetic organisms (Tian and Hua, 2010) or scavenge reactive oxygen species like singlet oxygen (^1^O_2_) and peroxyl radicals in non-phototrophic (Armstrong, 1997; Stahl and Sies, 2003; Slade and Radman, 2011). Carotenoids protect DNA from oxidative damage (Shahmohammadi et al., 1998), proteins from carbonylation (Tian et al., 2009), and membranes from lipid peroxidation (Sy et al., 2015). Previous research reports that the ability of oxygen quenching increases with the number of double bonds in the carotenoid molecule, yet quenching varies with chain length, structure, and functional groups (Hirayama et al., 1994). Conjugated keto groups and the presence of a cyclopentane ring increase quenching, while hydroxy, epoxy, and methoxy groups show lesser effects (Hirayama et al., 1994).

Currently, there are two well-established methods to detect and identify carotenoids; the first is detection and separation by HPLC (Tian et al., 2007) and the second is Raman spectroscopy. Raman spectroscopy is a spectroscopic technique used to observe vibrational, rotational, and other low-frequency modes in a molecule. It measures the spectrum of light scattered from a sample, which is irradiated with a monochromatic source in the visible, near-infrared, or UV region (Marshall et al., 2007). Carotenoids are π-electron-conjugated carbon chain molecules and are similar to polyenes with regard to their structure and optical properties (Marshall et al., 2007). Their strong color is due to an allowed π–π^∗^ transition that occurs in the visible region of the electromagnetic spectrum (Marshall et al., 2007). Carotenoids have two strong Raman bands due to in-phase ν_1_(C=C) and ν_2_(C–C) stretching vibrations of the polyene chain and a medium intensity feature due to in-plane rocking modes of CH_3_ groups attached to the polyene chain coupled with C–C bonds (Gill et al., 1970; Vítek et al., 2009). Because the Raman spectrum results from laser excitation giving rise to a series of characteristic bands in the range of 100–3,500 cm^-1^, the molecular signatures of biomolecules and minerals occur simultaneously in the same analytical interrogation process (Edwards et al., 2014).

One non-photosynthetic bacterium of significant astrobiological interest, *Deinococcus radiodurans*, is brightly colored due to the expression of six carotenoid pigments related to β-carotene, the dominant of which is deinoxanthin (Saito et al., 1998; Ji, 2010; Dartnell and Patel, 2014). Disrupting the synthesis pathway of deinoxanthin by a knock-out of the phytoene synthase *crtB* gene results in a colorless mutant which is more susceptible to oxidative DNA-damaging agents than the wild-type (Zhang et al., 2007). *D. radiodurans* is well known for its extreme resistance against prolonged desiccation, UV and ionizing radiation, oxidative stress, as well as genotoxic chemicals (Battista, 1997; Cox and Battista, 2005; Bauermeister et al., 2012). In particular, the high tolerance against ionizing radiation is perplexing, as there are no naturally occurring environments known that result in exposure exceeding 260 mGy per year (Ghiassi-nejad et al., 2002), making it unlikely that a species evolved mechanisms to protect itself against high dose ionizing radiation (Cox and Battista, 2005). A likely explanation for this extreme radiation tolerance has been suggested by Mattimore and Battista (1996), where the authors proposed that the radiation resistance is a consequence of an adaptation to prolonged desiccation, for example, desiccation, similar to γ-irradiation, introduces many DNA double-strand breaks into the genome of *D. radiodurans* (Cox and Battista, 2005). Alternatively, the radiation resistance may have evolved as an adaptation to permafrost or semi-frozen conditions where background radiation-induced DNA damage is accumulated or to high natural ionizing radiation levels in manganese-rich marine sediments (Richmond et al., 1999; Sghaier et al., 2007; Slade and Radman, 2011).

This resistance against desiccation and radiation makes *D. radiodurans* of high astrobiological interest to investigate the possibility of survival on arid planets such as Mars.

Presently, the International Space Station (ISS) is orbiting Earth in low-Earth orbit (LEO), providing scientists a unique opportunity to study the responses of terrestrial organisms to the space environment. Environmental parameters surrounding the ISS – such as vacuum (between10^-3^ and 10^-4^ Pa), intense solar, and cosmic radiation, as well as temperature extremes severely impact the survival of microorganisms (Horneck et al., 2012; Wassmann et al., 2012; Kawaguchi et al., 2013). Due to its resistance against radiation and desiccation, the resistance and response of *Deinococcus* spp. to simulated (Kawaguchi et al., 2013) or real space conditions (Dose et al., 1995) has been previously investigated. In the frame of the EXPOSE-R2 space mission (see Rabbow et al., 2017), *D. radiodurans* was exposed to simulated Mars conditions in LEO for 1.5 years as part of the Biology and Mars experiment (BIOMEX). For an in-depth description and goals of the BIOMEX investigations please refer to de Vera et al. (2012). Here we report on the stability and detectability of the biomarker deinoxanthin with Raman spectroscopy, the survival of *D. radiodurans*, and the genetic integrity of the 16S rRNA gene following exposure to Mars conditions, utilizing the space environment for a realistic simulation.

## Materials and Methods

### Cultivation

*Deinococcus radiodurans* DSM46620 was obtained from the Deutsche Sammlung von Mikroorganismen und Zellkulturen (DSMZ, Braunschweig, Germany). *D. radiodurans* Δ*crtB* was a kind gift from Prof. Chengxian Fang (College of Life Sciences, Wuhan University, Wuhan, China; Zhang et al., 2007). Both strains were routinely cultured in 2× TGY medium, composed of (per liter) 10 g tryptone, 6 g yeast extract, and 2 g d-glucose monohydrate, pH 7.2. For solid medium, 15 g/L agar was added to the nutrient solution before autoclaving. Organisms were cultured in 50 mL medium at 30°C, shaking at 200 *× g* for 2 days.

### Sample Preparation

After reaching late exponential phase (OD_600nm_ ∼1.0), samples were centrifuged for 15 min at 3,000 *× g* at 4°C and the resulting pellet was washed with 25 mL 1× phosphate-buffered saline (PBS), pH 7.4. Samples were centrifuged again for 15 min at 3,000 *× g* at 4°C and the resulting pellet was re-suspended in 3 mL 1× PBS. The cell concentration was determined in a Thoma Cell Counter and a working stock of 5 × 10^8^ cells/mL was prepared. Forty microliters of this solution was spotted onto quartz disks (ϕ9 mm, 1 mm thickness, 63.61 mm^2^ area) resulting in a final concentration of 2 × 10^7^ cells/disk. To evaluate possible beneficial effects of embedding cells in sulfatic Mars regolith simulant [S-MRS, composition and Raman spectroscopic properties of the simulant are described in detail by Böttger et al. (2012)], 10 mg/mL was added to the working solution. After this, the mixture was vortexed (Biovendis, IKA MS3 basic) for 10 s at full speed (3,000 *× g*) before each sample withdrawal to ensure comparable S-MRS distribution, and 40 μL was spotted onto quartz disks as described above. A detailed size distribution of the employed S-MRS is given by Baqué et al. (2015). The quartz disks were dried overnight in a clean safety workbench at room temperature (∼22°C and ∼40% relative humidity). All exposure experiments presented in this work have been conducted with desiccated organisms as described above.

### Electron Microscopy

To analyze the distribution of the simulated Mars regolith on the quartz disks, electron microscopy images were obtained. Samples were prepared as described above and 10 disks were evaluated with a Hitachi TM3000 Tabletop microscope with an acceleration voltage of 15 kV. Energy dispersive spectroscopy (EDS) analysis was performed with the program Quantax70 (Bruker).

### Extraction of Deinoxanthin

Cell extracts from *D. radiodurans* containing deinoxanthin were obtained according to a modified protocol of Lemee et al. (1997). Briefly, cells were grown until late-exponential phase (OD_600nm_ ∼1.0) and harvested by centrifugation for 20 min at 4,000 × *g*. Forty grams of cell pellet was mixed with 200 mL methanol containing 0.3 g of 2,6-di-*t*-*butyl*-*p*-cresol (BHT) as antioxidant, homogenized by adding 5 g of sterilized glass beads and vortexed for 10 min at 3,000 × *g*. The sample was then centrifuged for 10 min at 10,000 × *g* and the supernatant removed. This process was repeated four times until the pellet lost all visible color. Supernatants from this process were pooled and then concentrated via evaporation at 50°C for 24 h. Forty microliters was spotted onto similar quartz disks as previously described and the samples were dried overnight in a clean safety workbench at room temperature in darkness.

### Pre-flight Ground Tests

To determine the feasibility of the proposed experiments in space and to optimize sample preparation and analysis, three ground experiments were conducted before the mission, two experiment verification tests (EVTs) and one science verification test (SVT). The tested parameters for the EVTs were as follows: monochromatic UV-C_(254nm)_ radiation (NN 8/15; Heraeus Noblelight, Hanau, Germany) with fluences of 0, 10, 100, 1,000, and 10,000 J/m^2^, and temperatures of -25 and +65°C for 1 h, respectively. A second EVT investigated the effects of simulated solar radiation (achieved with a Wolfram halide lamp with a spectral irradiance in the range of 200–400 nm; Dr. Hoenle AG, Germany) with the following fluences: 0, 1.5 × 10^3^, 1.5 × 10^4^, 1.5 × 10^5^, 5.0 × 10^5^, and 8 × 10^5^ kJ/m^2^, respectively. The SVT consisted of the following parameters: Simulated Martian atmosphere (95.55% CO_2_, 2.70% N_2_, 1.60% Ar, 0.15% O_2_, ∼370 ppm H_2_O, pressure 10^3^ Pa; Praxair Deutschland GmbH) for 38 days with exposure to a simulated solar irradiation fluence of 1.5 × 10^6^ kJ/m^2^. To further simulate the space experiment conditions, samples were placed in three different compartments, which were stacked as: top (t) compartment, where samples were exposed to the full solar radiation spectra, middle (m) and bottom (b) where no solar radiation could penetrate the samples. This arrangement was also employed for the flight and a detailed description of the hardware is given by Rabbow et al. (2012).

### Flight Preparation and Mission Ground Reference (MGR) Preparation

Flight, MGR, and laboratory control samples were prepared from the same starting cultures. Flight and MGR samples were accommodated in the sample trays as described for the SVT test and laboratory controls were stored in the laboratory under ambient conditions (∼22°C and ∼40% relative humidity) in darkness until the flight sample returned. For flight samples, sample trays were accommodated in Tray II of the EXPOSE-R2 mission. Tray II was hermetically closed, evacuated, and flooded with 980 Pa pressure Mars gas (described above), and covered by a quartz window with transmission of solar electromagnetic radiation of λ > 200 nm, similar to the spectrum expected on the Martian surface. To attenuate the total mission UV fluence, samples were additionally covered with quartz neutral density filter (0.001% transmission). These filters were employed due to the results obtained during preliminary experiments (detailed in the results section). An identical arrangement of samples was used for the MGR experiment.

### Flight Parameters and Conditions

Samples were transported to Baikonur and launched on 23 August 2014 with the 56P Progress rocket. EXPOSE-R2 was installed on the URM-D platform on Svezda module on 18 August 2014 during the EVA-39 by Aleksandr Skvortsov and Oleg Artemyev. The cover, protecting samples from solar radiation before the start of the exposure period, was removed on 22 October 2014. Samples were outside the ISS for 534 days and exposed to solar radiation for 469 days. During the EXPOSE-R2 experiment, the height of the ISS varied between 398 and 417 km above Earth^1^. Over the duration of the mission, the upper surface of the sample trays was exposed to 549.88 MJ/m^2^ of solar radiation (200–400 nm). Due to the previously mentioned neutral density filters, the samples in the top layer were exposed to a fluence of 5.54 kJ/m^2^. The trays were returned to the interior of the ISS on 03 February 2016 and the BIOMEX trays arrived back on Earth on 18 June 2016 with the Soyuz 45S rocket. The total mission duration was 696 days from launch to return to ground. An identical arrangement of samples was used for the MGR experiment. During the MGR, the environmental data of the flight unit (e.g., temperature profile and UV exposure) were followed with a time-delay of 2 months within DLR’s Planetary and Space simulation facilities (Rabbow et al., 2016). Additional laboratory control samples from the same batch of bacteria were stored in the dark in a desiccator under ambient conditions in the laboratory until the end of the mission and analyzed in parallel to the flight samples.

### Sample Analysis

#### Raman Spectroscopy

After exposure to stress conditions, the detectability of deinoxanthin was determined by Raman spectroscopy. Sample disks were placed under a Raman Microscope (WITec), equipped with a 532 nm laser. Spectra were routinely obtained by employing 3.02 mW of laser power. Line scans were performed using the 10× optical objective and measured over a predetermined distance (10 μm) with 50 spectra accumulated with an integration time of 2 s. Each sample was analyzed by five line scans where the area of analysis was randomly chosen, resulting in a total of 250 scans per sample. Following the line scans, area scans were performed over a square of 50 × 50 μm^2^, divided into 25 lines per image with 25 points for each line measured and with 2 s integration time. All spectra (875 in total) were evaluated against predetermined quality criteria (**Figure 6**).

#### Survival

Following evaluation by Raman spectroscopy, the survival by cultivation was assessed by determining the number of colony forming units (CFUs). To remove cells from the quartz disks, the samples on the disks were coated with 30 μL 10% (w/v) polyvinylalcohol (PVA) and dried in a sterile safety cabinet at room temperature over-night. The solidified PVA was gently removed with flame-sterilized forceps and transferred into 500 μL 1× PBS (pH 7.4) buffer. This procedure was repeated three times with samples being stored at 4°C. Disks were inspected under the microscope (×60 magnification) to confirm that no residual samples were left. To investigate survival, 20 μL of a dilution series from the recovered samples was plated onto 2× TGY medium and incubated for up to 1 week at 30°C and evaluated daily for growth. The survival rate was calculated as relative survival after exposure to space conditions (*N*) compared with the untreated laboratory control (*N*_0_).

#### Integrity of 16S rRNA Gene

DNA from all samples was extracted using the XS-buffer method as previously described by Tillett and Neilan (2000), purified with a standard PCI (25:24:1) protocol, precipitated with ice-cold isopropanol and washed twice with 75% EtOH, and air-dried for 15 min. DNA was re-suspended in 30 μL dH_2_O and the concentration was determined with a Nanodrop spectrophotometer (Thermo Scientific, Wilmington, MA, United States) and 10 ng was routinely used as template for quantitative PCR (qPCR). Quantitative PCR was performed following exposure to space conditions and simulated Mars conditions by employing the forward primer Drad16S_F1 (5′-TTTATGGAGAGTTTGATCCTG-3′) and the reverse primer Drad16S_R1502 (5′-AAAGGAGGTGATCCAACC-3′) resulting in a 1,501 bp product. Primers were designed based on the available type strain sequence. Amplifications were performed in an Opticon2 system (BioRad) using the PeqLab KAPA Sybr FAST Kit. Thermal cycling conditions were: Initial denaturation at 94°C for 3 min, followed by 40 cycles of denaturation at 94°C for 30 s, primer annealing at 60°C for 20 s, and strand extension at 72°C for 90 s. The standard curve was calculated on the basis of a 16S rRNA gene product obtained from serial diluted (1:10; 1:100; 1:1.000; and 1:10.000), isolated DNA, obtained from a freshly grown WT culture. Melting curve analysis (0.2°C s^-1^) and agarose gel electrophoresis (2% agarose) revealed single amplicons for all samples (data not shown). The amplification efficiency (*E*) was calculated from the slope of the standard curve using the formula: *E*(%) = 10^(-1/slope)^-1 (Bustin et al., 2009). PCR efficiency and correlation coefficient for the standard curve were 81% and *r*^2^ = 0.998, respectively. Resulting values from the qPCR runs were converted to relative lesion frequencies per 1.501 kB (∼1.5 kB) DNA by application of the Poisson distribution (lesions/amplicon = -ln(*A*_t_/*A*_0_), where *A*_t_ represents the amplification of treated samples and *A*_0_ is the amplification of untreated controls) as previously described (Hunter et al., 2010).

## Results

**FIGURE 1:**
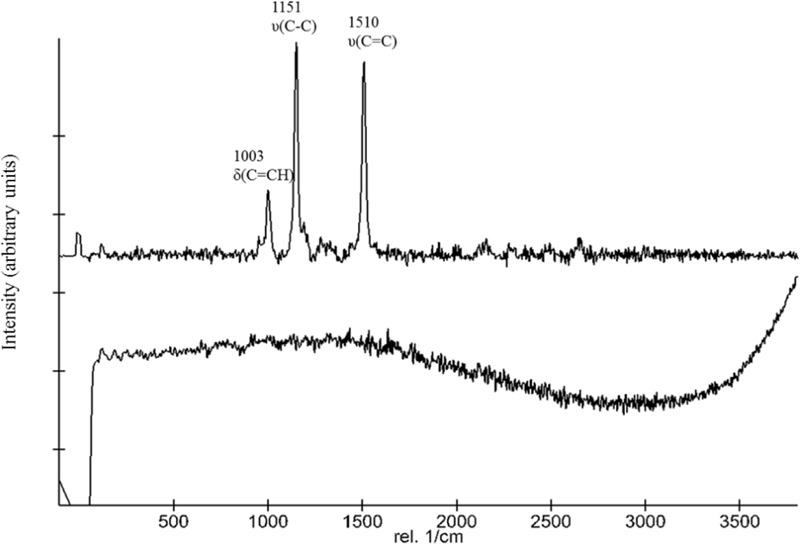
Comparison of Raman spectra of *D. radiodurans* and the *ΔcrtB* mutant. The top spectrum was obtained from *D. radiodurans* and the bottom spectrum from the *ΔcrtB* strain. The top spectrum shows the distinctive 1,003, 1,151, and 1,510 cm^-1^ peaks of deinoxanthin.

The suitability of Raman spectroscopy to detect carotenoids is well established. **Figure 1** shows representative stacked Raman spectra from the two strains employed in this study. The spectra of the WT contain major features at approximately 1,003, 1,151, and 1,510 cm^-1^. The bands at 1,510 and 1,152 cm^-1^ are due to in-phase C=C (*ν*_1_) and C–C stretching (*ν*_2_) vibrations of the polyene chain in carotenoids. Additionally, in-plane rocking modes of CH_3_ groups attached to the polyene chain coupled with C–C bonds occurred in the 1,003 cm^-1^ region. The spectra obtained for the *ΔcrtB* strain show no distinctive features or peaks, confirming a lack of deinoxanthin or any other Raman active compound under the tested conditions (**Figure 1**). No significant interference by the S-MRS was detected.

**FIGURE 2:**
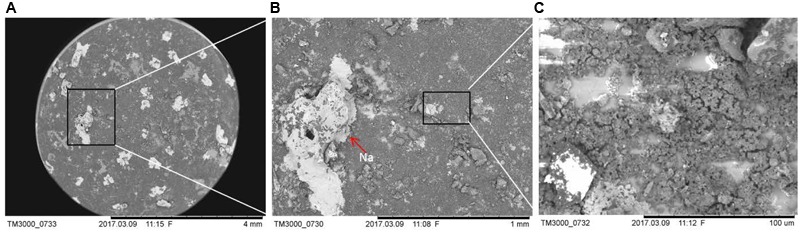
Electron microscopic evaluation of the distribution of the simulated Mars regolith on the quartz sample disks. Image **(A)** was taken with ×25, **(B)** with ×1,000, and **(C)** with ×1,500 magnification, respectively. Elementary analysis was conducted by EDS.

**FIGURE 3:**
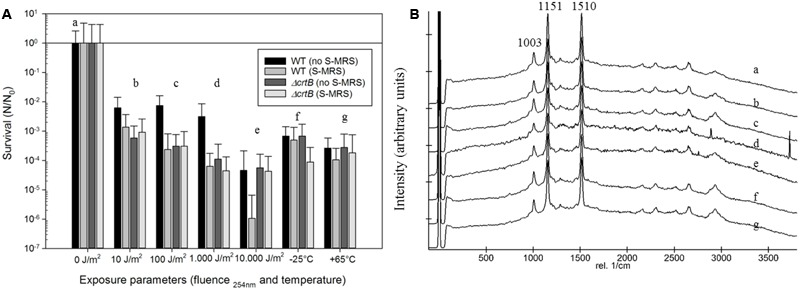
**(A)** Survival of *D. radiodurans* and the *ΔcrtB* strain following exposure with or without the protection of simulated Martian regolith to UV-C_254nm_ radiation, different temperatures, and simulated Mars-like solar radiation. The survival is given by the quotient *N*/*N*_0_, where *N*_0_ is the number of colonies of the non-treated samples and *N* that of the exposed samples to the different experimental conditions. **(B)** Stacked Raman spectra from *D. radiodurans* exposed in the presence of regolith following respective treatments. Small letters (a–g) correspond to the treatment in **(A)**.

**FIGURE 4:**
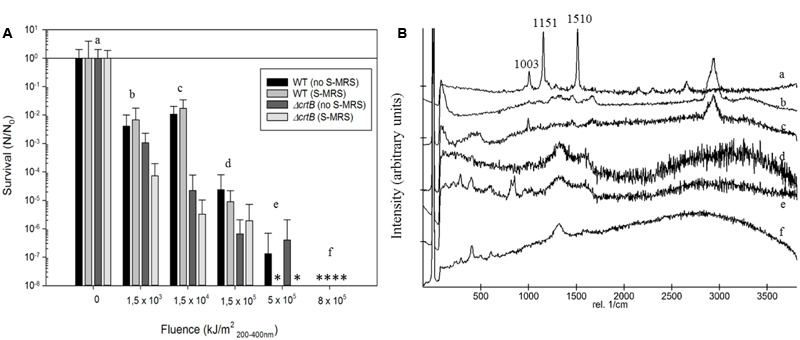
**(A)** Survival of *D. radiodurans* and the *ΔcrtB* strain following exposure to simulated solar radiation up to 8 × 10^5^ kJ/m^2^. ^∗^ indicates that no survival was observed. The survival is given by the quotient *N*/*N*_0_, where *N*_0_ is the number of colonies of the non-treated samples and *N* that of the exposed samples to the different experimental conditions. **(B)** Stacked Raman spectra from *D. radiodurans* exposed with regolith following respective treatments. Small letters (a–f) correspond to treatment in **(A)**.

To evaluate if *D. radiodurans* and the *ΔcrtB* mutant are suitable candidates for the scientific questions asked by the BIOMEX proposal, several ground tests were conducted. In particular, the stability of deinoxanthin as a biomarker was of higher interest than the survival of the cells. Evaluations of the surface properties of the dried samples combined with the simulated Mars regolith revealed inhomogenic coverage of the quartz disks with cracks within the layers (**Figure 2**). Experiment verification tests were designed to evaluate if the tested organism survive extreme environments such as radiation, desiccation, and vacuum as foreseen in the space experiment. A SVT was designed to simulate the complete mission in a shorter timeframe on Earth, to evaluate if the chosen organism has the potential of survival, and as a rehearsal for the procedures and logistics. The first EVT focused on the survivability of both strains exposed to monochromatic UV-C_(254nm)_ radiation as well as temperature extremes as they may occur during the space experiment and the results are given in **Figure 3**. The second EVT focused on the survival following exposure to simulated polychromatic solar UV radiation with the biologically deleterious spectral irradiance calculated between 200 and 400 nm and results are presented in **Figure 4**. The survival of *D. radiodurans* and the *ΔcrtB* mutant displayed a dose-dependent decrease of survival when exposed to germicidal UV-C_(254nm)_ radiation. A reduction of survival by three orders of magnitude was observed when both strains where exposed to high/low temperature. The addition of simulated Martian soil showed no significant positive or detrimental effect on the survival of both strains under the chosen conditions. Following all exposure experiments, the detectability of the deinoxanthin was evaluated and is shown in **Figure 3B**. No reduction or shifts in the Raman signal were observed following the described treatment. Similar to exposure to UV-C radiation, exposure to the simulated solar spectrum lead to a dose-dependent decrease in survival and growth was undetected in the timeframe used following exposure up to 8 × 10^5^ kJ/m^2^ (**Figure 4B**). Again, the addition of simulated Martian soil provided no additional protection. Compared to UV-C_(254nm)_ radiation, deinoxanthin is strongly susceptible to simulated polychromatic radiation (**Figure 4B**). Although the cells survived simulated polychromatic light up to 1.5 × 10^4^ kJ/m^2^, the previously seen signature peaks of deinoxanthin were no longer detectable by Raman spectroscopy following exposure to 1.5 × 10^3^ kJ/m^2^ and higher.

**FIGURE 5:**
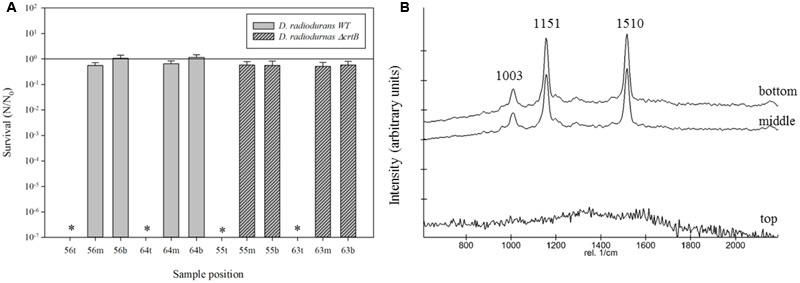
**(A)** Survival of *D. radiodurans* and *ΔcrtB* with regolith following the science verification test (SVT). ^∗^Indicates that no survival was observed. **(B)** Stacked Raman spectra from samples exposed to solar irradiance (top), and no irradiance (middle and bottom).

Results from the science verification test (SVT) are given in **Figure 5**. It is evident from the results that cells in the top layer (exposed to the full solar spectrum), did not survive. This could also be confirmed by the Raman spectra which show no characteristic Raman signals of the WT (**Figure 5B**). Organisms in the middle and bottom compartment without any exposure to UV radiation survived without any significant loss in viability and deinoxanthin was easily detected. For the reason that both, the cell survival and the Raman signature, are highly affected by polychromatic light, we chose a strong neutral density filter (0.001% T), to reduce the fluence over the duration of the mission. Following the previously mentioned measured fluences, samples were exposed to 5.54 kJ/m^2^ solar radiation (200–400 nm) during the duration of the mission.

**FIGURE 6:**
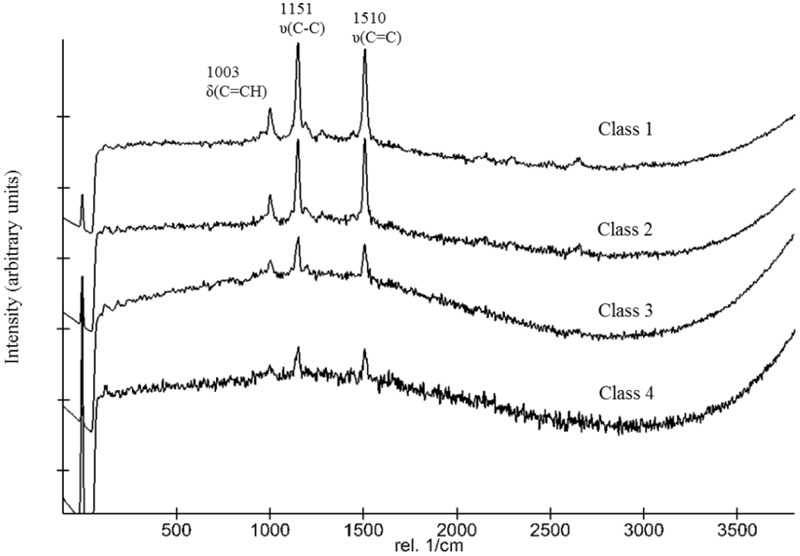
Categorization of spectra and classification in different classes.

**FIGURE 7:**
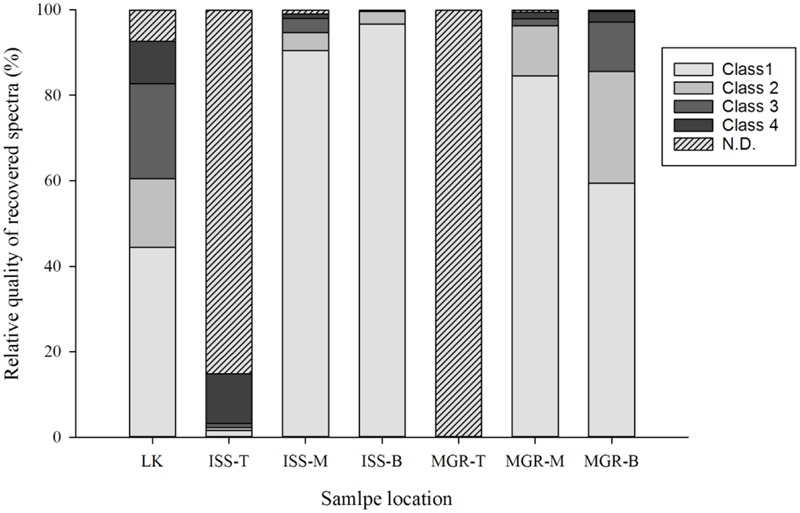
Evaluation of the deinoxanthin signal intensity according to the defined classes (**Figure 6**) following ground simulations and samples exposed to outer space conditions during the EXPOSE-R2 mission. N.D. denotes that no signal could be determined. Eight hundred and seventy-five spectra were evaluated for each bar and scored according to the previously determined signal class (**Figure 6**). Sample location indicates whether samples were exposed to outer space (ISS) or part of the mission ground reference (MGR). LK denotes a laboratory control from the same batch that was sent to the ISS. T, M, and B describe the sample position within the tray as follows: T, top; M, middle; and B, bottom. Only the top samples were exposed to solar radiation.

**FIGURE 8:**
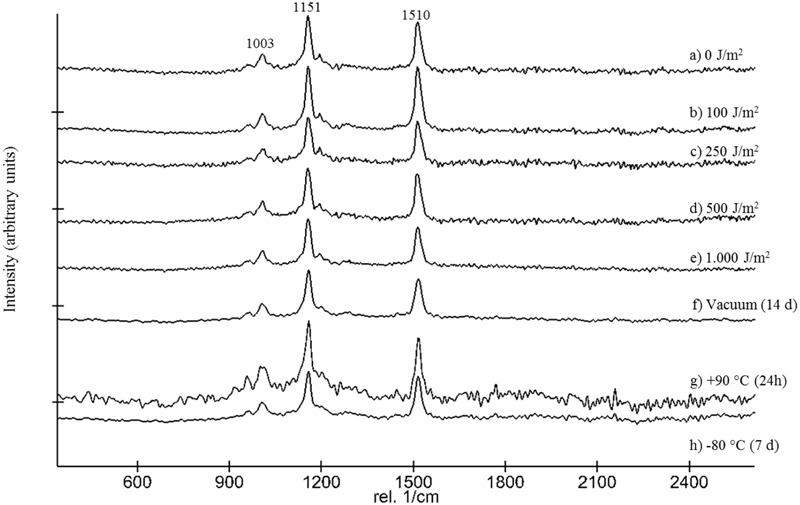
Stacked Raman spectra showing the effect of UV-C_254nm_ radiation up 1,000 J/m^2^ (a–e), vacuum (f), as well as +90°C (g) and –80°C (h) to MeOH/BHT extracted cell homogenate of *D. radiodurans*.

Following the return of the samples and the arrival in the laboratory, survival was evaluated immediately and Raman spectra were obtained from all samples. *D. radiodurans* grows best between 30 and 37°C, with a doubling time of 1.5–3 h (Cox and Battista, 2005). Unfortunately, no survival was observed for either the WT or the *ΔcrtB* mutant, even after prolonged incubation for up to 3 weeks at 30°C. Given roughly 21× longer to grow than a strain maintained under laboratory conditions, we believe that the probability of observing growth from the cells exposed to space conditions is very low. Expectedly, evaluation of the 16S rRNA integrity revealed that the 16S rRNA gene suffered severe damage during this process. Results from the laboratory control show that the prolonged desiccation and storage resulted in an 81% chance of a lesion within the 16S rRNA gene, without being exposed to solar radiation or other detrimental factors (**Table 1**). The stability of deinoxanthin was investigated by Raman spectroscopy and accumulated spectra were divided into four different classes (**Figure 6**). Spectra of classes 1 and 2 show a strong signal/low noise ratio with the three characteristic peaks dominating the spectra. Class 3 spectra have a medium signal/noise ration with the peaks fading. Class 4 spectra are classified by a weak signal/noise ratio with the peak at 1,003 cm^-1^ scarcely detectable. Results of this evaluation are given in **Figure 7**. For all flight and MGR samples, 875 spectra were obtained and evaluated separately. Samples exposed to solar radiation lost the deinoxanthin signal almost completely, with samples exposed in the space mission marginally better preserved than samples from the MGR (**Figure 7**). Samples not exposed to solar radiation revealed strong deinoxanthin signals, both after spaceflight and ground simulation. It is interesting to note that the signal from the laboratory control (kept in dark) appears to be significantly weaker (44.1% class 1 spectra, compared to 96.7% class 1 spectra from the ISS bottom sample) compared to spectra from samples not exposed to solar radiation during the space mission. To investigate if deinoxanthin is resistant to detrimental environmental conditions when not protected by the cell, it was extracted from cell homogenate, using methanol and BHT, and the resulting concentrated sample was exposed to UV-C radiation, vacuum, and different temperature extremes (**Figure 8**). Exposure to heat, (+90°C) for 24 h, resulted in a visible quality loss of the spectrum. All other tested factors did not influence the quality of the obtained spectra significantly.

**Table 1:** Relative lesion frequency in the 16S rRNA gene fragment (1.5 kb) calculated against DNA extracted from freshly grown *D. radiodurans*.

Sample location	Relative lesion frequency (%)	Sample location	Relative lesion frequency (%)
LC WT	81 (±2)	LC *ΔcrtB*	76 (±5)
ISS WT_t	100	ISS *ΔcrtB*_t	100
ISS WT_m	100	ISS *ΔcrtB*_m	65 (±3)
ISS WT_b	94 (±6)	ISS *ΔcrtB*_b	70 (±2)
MGR WT_t	100	MGR *ΔcrtB*_t	100
MGR WT_m	93 (±5)	MGR *ΔcrtB*_m	83 (±6)
MGR WT_b	89 (±1)	MGR *ΔcrtB_b*	81 (±3)

*Sample location indicates whether samples were exposed to outer space (ISS) or part of the mission ground reference (MGR). LC denotes a laboratory control from the same batch that was sent to the ISS. Lowercase t, m, and b describe the sample position within the tray as follows: t, top; m, middle; and b, bottom. Only the top samples were exposed to solar radiation.*

## Discussion

The BIOMEX was aimed at exposing different extremophilic organisms and potential biomarkers to Martian-like conditions in LEO, being simulated in one of the trays as part of the EXPOSE-R2 mission. Here we report the results of the survival and genetic integrity of *D. radiodurans* and the *ΔcrtB* mutant and the stability and detectability of deinoxanthin following the exposure. The main focus of this work was to determine the stability of deinoxanthin and the possibility of this molecule to be a useful biomarker to detect life on other planets. Although both strains survived the preliminary tests relatively well, except intense exposure to solar radiation, we were not able to recover colony-forming units from any of the samples, not even the laboratory control. It is well established that *D. radiodurans* is desiccation resistant (Cox and Battista, 2005; Bauermeister et al., 2011); however, desiccation for 17 months without protective substances such as glucose and storage in a non-oxidizing atmosphere such as argon lead to a lethal amount of DNA double-strand breaks as previously reported by Dose et al. (1995). Similar to these results, storage of dried *D. radiodurans* and the *ΔcrtB* mutant strain in the dark under ambient laboratory conditions lead to an 81% chance of a lesion within the 16S rRNA gene (**Table 1**). Exposure to extraterrestrial Mars-like solar UV radiation led to a 100% probability of a lesion (**Table 1**). The addition of the simulated Martian regolith did not improve the survivability of the strains, although a beneficial effect has been previously reported (Pogoda de la Vega et al., 2007); however, our results suggest that a long-term storage of cells embedded in the dust has a detrimental effect on the cells. A possible explanation for this conundrum may be that UVR-induced radicals form in Mars substrates (Shkrob et al., 2010), which might be mimicked by the employed S-MRS and therefore may affect the survivability (Meeßen et al., 2015). It has previously been reported that even multilayers of cells act as a protective barrier for the cells underneath (Kawaguchi et al., 2013); here, however, the impact of prolonged desiccation was sufficient to kill the cells even without exposure to solar radiation.

The picture is different when investigating the stability and detectability of deinoxanthin by Raman spectroscopy. Carotenoids serve as accessory pigments to increase the efficiency of photosystems but are also synthesized by many non-photosynthetic bacteria as they function as efficient scavengers of ROS (Dartnell et al., 2012). It has previously been shown that deinoxanthin is particularly effective in scavenging H_2_O_2_ and singlet oxygen, performing better than other carotenes or xanthophylls (Tian et al., 2007; Dartnell et al., 2012). Furthermore, deinoxanthin is also remarkably stable when not inside the cell, compared to other possible biomarkers such as DNA, which is more vulnerable against radiation when not protected by the cell (Leuko et al., 2011). The abilities of Raman spectroscopy to detect biomarkers such as carotenoids from samples collected in remote and extreme areas such as the Atacama Desert, Death Valley, volcanic rocks, or Antarctica have been extensively tested and verified (Edwards et al., 2005; Jorge Villar et al., 2006; Vítek et al., 2010; Winters et al., 2013). It was also shown that carotenoid signatures can be recovered from cryptochasmoendoliths, preserved microbial filaments, and relict sedimentary structures (Edwards et al., 2007). However, we also know from previous research that carotenoids are vulnerable toward oxidation and photodecomposition (Vítek et al., 2014). The detrimental effect of solar radiation on pigmentation is well established and has been previously investigated for *Cyanophora paradoxa* (Häder and Häder, 1989) or for corals (Brown and Dunne, 2008). This photodegradation is primarily caused by the UV-A part of the solar spectrum (Kumar et al., 2015); however, a destruction of Raman biosignatures is also commissioned by high doses of ionizing radiation (Dartnell et al., 2012) and γ-irradiation (Meeßen et al., 2017). The detrimental effect of radiation has also been demonstrated by previous space missions to LEO, where Cockell et al. (2011) report the complete disappearance of previous β-carotene Raman signatures from several phototrophic organisms. Over the history of Mars, galactic cosmic radiation and solar cosmic rays played an important role in the degradation of organic molecules near the planetary surface, should they have ever been present (Vítek et al., 2014). Calculations by Pavlov et al. (2012) suggest that organic molecules with masses greater than 100 amu (atomic mass unit) would be destroyed in less than 1 billion years in the top 5 cm of Martian regolith (Vítek et al., 2014). Furthermore, analysis by Raman spectroscopy of Martian meteorites, e.g., MIL03346 which belongs to the nakhlite group, failed to identify any biological signature (Wang et al., 2015). However, only a diminutive amount of Martian meteorites has been analyzed, so future research may reveal signs of biological life in a meteorite.

Our results presented here suggest that 1.5 years exposure to the Mars-like solar UV spectrum is already sufficient to degrade deinoxanthin beyond detectability with Raman spectroscopy, at the investigated total fluence. To search for signs of extinct life on the surface of Mars seems therefore pointless; however, below the surface the picture may be completely different. Protected from the most detrimental environmental source, radiation, signs of earlier life may still be preserved. Caves, or other subterranean cavities, can be suspected on other planets and in particular Moon or Mars show clear photographic evidence of lave tube caves (Boston et al., 2003).

In this study we showed the successful detection of the carotenoid deinoxanthin following exposure to Mars conditions for 1.5 years simulated in space when protected from solar radiation. Even though some signals were recovered from samples exposed to solar radiation, the vast majority of carotenoids were degraded beyond detectability by Raman spectroscopy. All other tested space relevant conditions, such as temperature oscillations, vacuum, or a Martian atmosphere, had no detectable effect on the molecule. Future missions are planned to investigate caves on Mars and by extending our search for life on Mars to the subsurface, we certainly would increase our chances to find traces of extant or extinct life on our neighbor planet.

## Author Contributions

SL and MB performed the experimental setup and the survival assays; SL, MB, UB, FH, and J-PdV performed Raman spectroscopic analysis; ER and AP conducted the MGR experiments; AP conducted Electron microscopy. SL, MB, FH, UB, ER, AP, PR, and J-PdV helped with data interpretation, scientific guidance, and preparation of the manuscript.

## Conflict of Interest Statement

The authors declare that the research was conducted in the absence of any commercial or financial relationships that could be construed as a potential conflict of interest.

## Footnotes

1 http://www.heavens-above.com/IssHeight.aspx
